# Factors affecting genetic transformation by particle bombardment of the prickly pear cactus (*O. ficus-indica*)

**DOI:** 10.1007/s13205-019-1627-6

**Published:** 2019-02-20

**Authors:** Paola Isabel Angulo-Bejarano, Ashutosh Sharma, Octavio Paredes-López

**Affiliations:** 1 Centro de Investigación y de Estudios Avanzados-IPN, Unidad Irapuato, Km 9.6 Libr. Norte Carr. Irapuato-León, Apdo. Postal 629, Irapuato, 36824 Guanajuato, Mexico; 2 Tecnologico de Monterrey, School of Engineering and Sciences, Epigmenio González No. 500 Fracc. San Pablo, 76130 Queretaro, Queretaro Mexico

**Keywords:** Cactus, Genetic transformation, Biolistics, Biotechnology

## Abstract

In the present study, a novel transformation protocol for *Opuntia ficus-indica* was generated by means of particle bombardment. The best conditions obtained were: 900 psi rupture disk pressure, 8 cm microprojectile travel distance, and 4 h of exposition to 0.2 M mannitol. For all experiments, gold particles coated with 1.0 µg/µL of pBI426 plasmid DNA were used. With all these conditions, a 23% of transformation efficiency in terms of regeneration in selection media (100 mg/L kanamycin) was obtained. Interestingly, the presence of both transgenes: *nptII* and *uidA*, by means of PCR and RT-PCR assays was detected. The regeneration percentage achieved in terms of stable integration for both genes was 10%. In addition, we also detected adequate amounts of *β*-glucuronidase activity by means of the GUS fluorometric assay. The procedure described in the present investigation reveals the feasibility of using nopal for the introduction, expression, and possible production of heterologous proteins.

## Introduction

Nopal (*O. ficus-indica*) belongs to the Cactaceae family, sub-family Opuntioideae, and constitutes the cactus species with the highest economic importance worldwide. It is grown in America, Africa, Asia, Europe, and Oceania (Angulo-Bejarano and Paredes-López 2012). The number of species belonging to the *Opuntia* genus may range between 160 (Gibson and Nobel 1986) and 250 (Britton and Rose 1963) and most of them are thought were originated in Mexico. At present, it is considered important for its use as food and forage, and as a source for nutraceuticals (Angulo-Bejarano et al. 2014).

Nopal plants are able to thrive under extremely contrasting environmental conditions, such as poor soils with low pH (Blanco et al. 2003). In addition, they are highly efficient in terms of water usage, mainly due to their CAM metabolism (Herrera 2009). Nopal plants are also characterized for a slow growth due to a long juvenile phase in comparison with asexually propagated material (Silos-Espino et al. 2006). The main meristematic tissue arises from structures called areoles which cover the stems, and that can be stimulated or activated during most in vitro tissue protocols in nopal (Angulo-Bejarano and Paredes-López 2011).

The introduction of new traits in the prickly pear cactus relies on the establishment of gene transfer technologies. In fact, the use of such technologies may aid for increasing nutritional value or in the production of nutraceuticals. A reliable plant genetic transformation system is imperative to allow the use of nopal as a plant bioreactor for the production of commercial or therapeutic molecules. In fact, nopal is a potential candidate to be used as plant bioreactor system due to its capacity to thrive in contrasting environmental conditions and to its high water usage efficiency, among other possibilities (Angulo-Bejarano 2013).

The feasibility for heterologous DNA integration in nopal was formerly described by our research group in 2006, where explants from *O. ficus-indica* cv. “Villanueva” were utilized and stably transformed by means of *Agrobacterium tumefaciens* (Silos-Espino et al. 2006). This report demonstrates, apparently for the first time, the use of prickly pear cactus in the expression of recombinant proteins. However, several attempts had been made previously to achieve genetic transformation through particle bombardment in *O. ficus-indica*. In 1998, Llamoca-Zarate et al. reported particle bombardment on friable calli of nopal cell suspensions. The transient expression of *uidA* and *nptII* genes was described, but no stable integration of these genes was registered. In a second attempt, they reported the transient expression of the *uidA* gene in shoot apical meristems from nopal without evidence for stable foreign DNA integration (Llamoca-Zarate et al. 1999a, b). Another study depicted particle bombardment in nopal shoot apical meristems that were transiently transformed to express the *uidA* and *Atahas* gene. Nevertheless, no stable integration of the transgene was described (Cruz et al. 2009). Therefore, the aim of the present study was to establish the most suitable conditions for particle bombardment in nopal by means of transient GUS expression and to assess the stable integration of foreign DNA by the molecular analyses.

## Materials and methods

### Materials

For all experiments *O. ficus-indica* cv. “Blanco sin Espinas”, a commercial variety that was kindly provided by PRONOPVAL at Valtierrilla, Guanajuato was used. The cladodes were disinfested and micropropagated according to García-Saucedo et al. (2005). In addition, to validate the transformation process, leaf explants from micropropagated *Nicotiana tabacum* var. Xanthi were used as a control. Tobacco internode micropropagation was done according to the method reported by Camacho-Beltrán (2008).

**Fig. 1:**
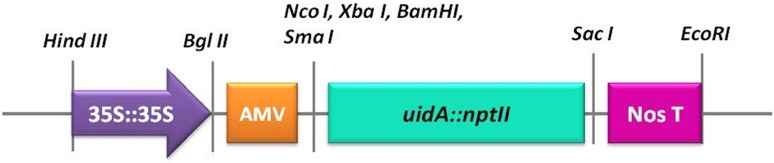
Schematic representation of a fragment of pBI426 plasmid used for nopal genetic transformation, adapted from Datla et al. (1991). Double CaMV 35S promoter; a leader sequence from alfalfa mosaic virus (AMV), a transcriptional fusion between *uidA* and *ntpII* genes, and the *nos* terminator

For the genetic transformation process, we utilized the pBI426 plasmid which was first described in Datla et al. (1991) **(**Fig. 1**)**. The plasmid DNA was isolated using the PureLink™ Quick Plasmid Miniprep Kit from Invitrogen, following the manufacturer′s protocol. The plasmid DNA obtained was quantified in a Nanodrop® NE-1000 equipment and analyzed in 0.8% agarose gel stained with ethidium bromide.

### Preparation of microcarriers

#### Tungsten particles

Sixty milligrams of tungsten particles (M-10) were re-suspended in 1.5 mL of 0.1 N HNO_3_ and processed according to the protocol described by Camacho-Beltran (2008).

#### Gold particles

Sixty milligrams of gold particles (0.6 µm, Biorad^®^ Hercules, CA. USA) were prepared according to Daniell et al. (2005).

## Establishment of the ideal conditions for particle bombardment in nopal explants, by transient GUS expression

Nopal micropropagated plantlets were cut in approximately 1 cm^2^ explants and placed carefully in the center of a Petri plate (60 × 15 mm) avoiding the presence of empty spaces between them due to the irregular morphology of explants. Four explants were used per bombardment event. Each Petri plate contained the osmotic medium which, for the first experiments (testing helium rupture disk pressure, distance of flight, time of exposure, DNA concentration, and type of particle) contained MS (Murashige and Skoog 1962) basal salts supplemented with maltose 15% v/v adjusted to pH 5.7 and solidified with 7 g/L agar.

The effect of rupture disk pressure and microprojectile distance was determined by analyzing three different rupture disk pressures: 450, 900, and 1350 psi, versus three distances (8, 11, and 14 cm) of flight allowed by the particle bombardment gun. The time of exposure to the osmotic agent previous to particle bombardment was adjusted; thus, the effect of 4, 6, and 8 h was examined. In addition, we evaluated the effect of DNA concentration loaded into the microcarriers; therefore, the use of 0.5, 1, and, 1.5 µg/µL was tested. In addition, we evaluated the effect of using either tungsten or gold particles. Finally, the effect of different osmotic agents (maltose, mannitol, sorbitol, and their combinations, all at a concentration of 0.2 M) was assessed. In all cases, tobacco leaf explants were used as positive control.

After each biolistic event, the explants were kept under dark conditions at 28 ± 2 °C to allow for their recovery. Then, they were analyzed for transient GUS expression by the histochemical staining method reported by Jefferson et al. (1987). Blue spots or stains (*foci*) were considered as positive *uidA* gene integration events and were considered as an expression unit, which was registered numerically (Hagio et al. 1995).

### Statistical analysis

Data were analyzed using one-way analysis of variance (ANOVA). The means were compared with Tukey’s Honestly Significant Difference (HSD) at *α* = 0.05. Only in the case of the type of osmotic medium, we used Duncan’s test at *α* = 0.05. For all analyses, the software Statgrafics CENTURION XV (Statistical Graphics, Co) was used.

#### Stable nopal transformation by particle bombardment

After bombardment, the explants were allowed to recover under dark conditions as described previously. Next, they were transferred to the indirect organogenesis regeneration medium (Angulo-Bejarano and Paredes-López 2011). MS supplemented with 0.5 g/L 2,4-D and 0.5 g/L BA, pH 5.7 solidified in 7 g/L agar without a selecting agent during the first 15 days. After this period, they were transferred to MS medium supplemented with 50 mg/L kanamycin (15 days) and 100 mg/L (30 days) where they were kept until the end of the experiment. In all cases, a photoperiod of 16 h light/8 h darkness and 28 ± 2 °C was used. Transformation efficiency in terms of the survival in the selection medium was established as the number of green, healthy and sprouting explants in selection medium related to the total number of bombarded explants.

#### Molecular analysis

Total genomic DNA was isolated from resistant explants of putatively transformed plants according to Angulo-Bejarano (2013) and used to detect the presence of the *uidA* and *nptII* transgenes by polymerase chain reaction (PCR). To amplify a 617 bp fragment of the *nptII* gene, we utilized the following set of primers: ***nptII*** **forward** 5′-TATTCGGCTATGACTTGGGC-3′ and ***nptII*** **reverse** 5′-GCCAACGCTATGTCCTGATA-3′. In addition, to amplify a 534 bp fragment of the *uidA* gene, the following set of primers: ***uidA*** **forward** 5′- CGTCCTGTAGAAACCCCAAC-3′ and ***uidA*** **reverse** 5′CGGCGTGGTGTAGAGCATTA-3′ were used.

Total RNA was extracted according to the method of Valderrama-Chairez et al. (2002). Approximately 0.5 g of fine-ground nopal tissue previously frozen in liquid nitrogen was used for the extraction procedure. The total RNA extracted was used for RT-PCR analysis. A range of 1–3 µg of total RNA from all samples was used for c-DNA synthesis. To validate the quality of the c-DNA obtained, a 500 bp fragment of the actin gene was amplified (data not shown). The c-DNA obtained was used as template to amplify the same fragments for *nptII* and *uidA* genes.

β-glucuronidase expression, utilizing the fluorometric assay described by Jefferson et al. (1987), was also analyzed. Approximately 100–200 mg of fine-ground tissue previously frozen in liquid nitrogen, from putatively transformed and untransformed plants, was used for this assay. Protein quantification was done utilizing the BCA™ Protein Assay Kit (Pierce, Rockford, IL, USA). 4–10 µg of protein extract were mixed with the GUS Assay Buffer; all readings were done with a fluorometer (Hoefer DyNA Quant 200; Pharmacia Biotech, Golden Valley, MN, USA). A commercial β-glucuronidase (Sigma-Aldrich, St. Louis MO, USA) was used as standard for all the readings.

Transformation efficiency in terms of the molecular analyses was expressed as the number of explants that were PCR, RT-PCR, and GUS fluorescent positive versus the overall bombarded explants.

## Results

### Particle bombardment conditions for *Opuntia* explants

**Fig. 2:**
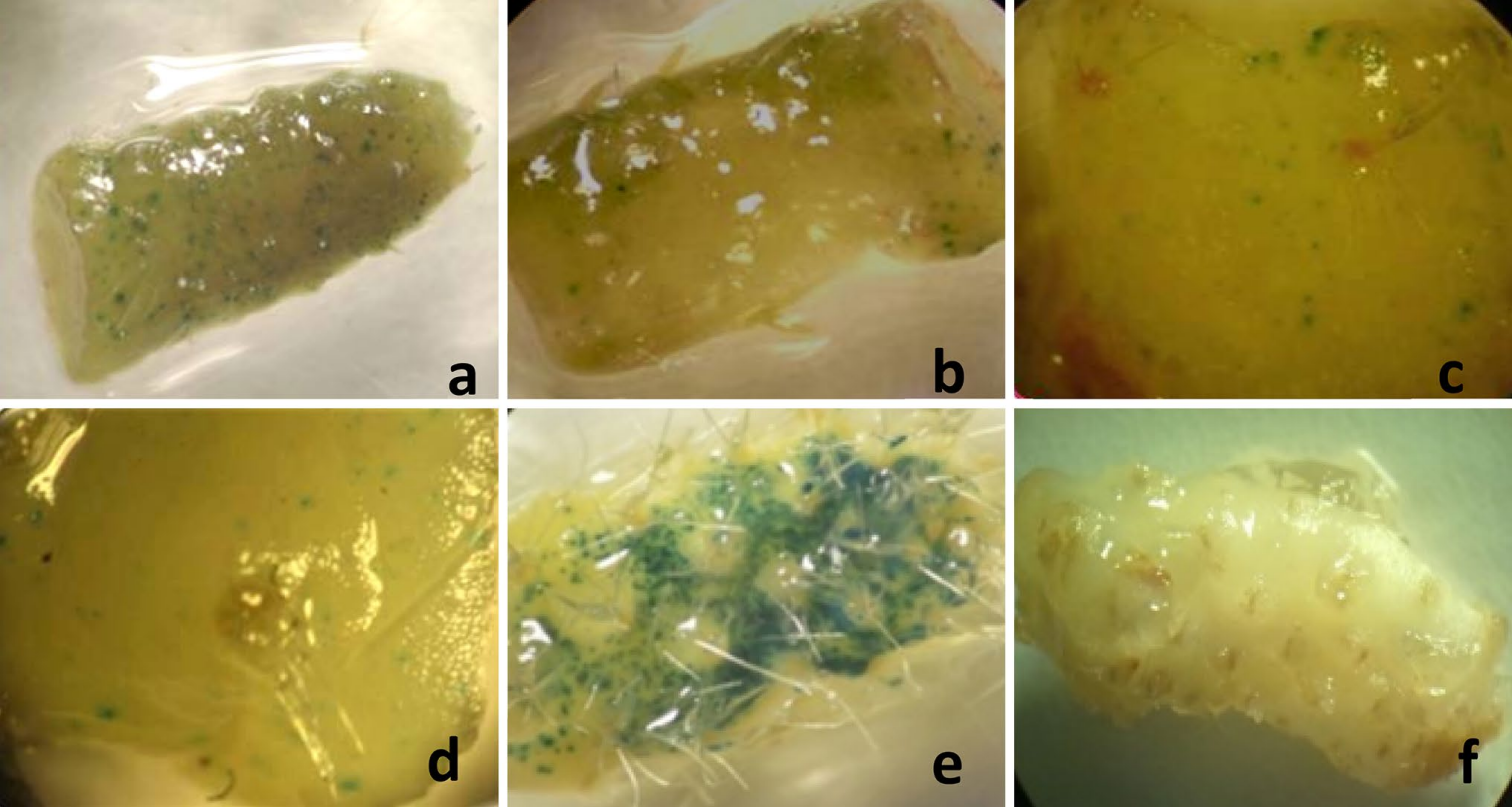
Results of the adjustment of particle bombardment conditions for nopal explants by means of transient *uidA* expression. **a** 900 psi and 8 cm distance of flight, **b** time of exposition to the osmotic agent (4 h), **c** plasmid DNA concentration (1 µg/µL), **d** type of microcarrier (gold); **e** type of osmotic agent (mannitol 0.2 M), **f** untransformed nopal used as negative control for endogenous GUS expression

The best particle bombardment conditions were established by means of analyzing the transient GUS expression in 1 cm^2^ micropropagated nopal explants. As a first condition, we evaluated the effect of helium pressure (450, 900, and 1350 psi) versus the distance of flight (8, 11 and 14 cm) **(**Table 1**)**. We found statistical differences among the helium pressures analyzed (α = 0.05) 900 psi being the most adequate, with the highest *foci* formation levels. No statistical differences were found between the use of 450 and 1350 psi. On the other hand, significant statistical differences (α = 0.05) were found among the distances of flight analyzed; 8 cm was the most appropriate, since it allowed for the highest foci formation in nopal explants (Table 1; Fig. 2a).

**Table 1:** Establishment of conditions for particle bombardment in *O. ficus-indica* explants through transient expression

Pressure (Psi)	Distance (cm)	Foci number (mean ± SEM)*
Pressure and distance of flight		
450	8	0.66 ± 0.14^b^
	11	0^b^
	14	0^b^
900	8	18.83 ± 4^a^
	11	0.47 ± 0.13^b^
	14	0^b^
1350	8	4.28 ± 0.76^b^
	11	0.96 ± 0.21^b^
	14	0.97 ± 0.28^b^
Exposition time (h)		
4		38 ± 0.44^a^
6		8.5 ± 0.08^b^
8		7 ± 0.26^b^
DNA concentration (µg/µL)		
0.5		7.46 ± 1.02^b^
1.0		23.93 ± 3.55^a^
1.5		14.46 ± 1.8^ab^
Particle type		
Gold		53.14 ± 0.29^a^
Tungsten		29.16 ± 0.21^b^
Osmotic medium (0.2 M)**		
Mannitol		197 ± 6.7^a^
Sorbitol		137 ± 4.5^b^
Maltose		0^c^
Maltose/sorbitol		25 ± 3.2^c^
Mannitol/maltose		21 ± 1.63^c^
Mannitol/sorbitol		0 ± 0.125^c^
Mannitol/sorbitol/maltose		2.3 ± 0.0^c^

*Means with same letter are not significantly different according to Tukey′s Honestly Significant Difference (HSD) at *α* = 0.05

**Means with the same letter are not significantly different according to Duncan′s test at *α* = 0.05

The effect of the exposition time to an osmotic agent, previous to particle bombardment, was also analyzed. For these first adjustments, we utilized maltose 15% (v/v). According to Tukey’s HSD test, statistical differences (α = 0.05) were found between the use of 4 h and the rest of the exposition times analyzed (Table 1; Fig. 1b). Similarly, we evaluated the effect of plasmid DNA concentration loaded in tungsten particles. According to our findings, we observed a statistically significant difference (*α* = 0.05) between the use of 1 µg/µL and the rest of the evaluated concentrations (Table 1; Fig. 2c). Next, we analyzed the effect of particle type (tungsten and gold particles). Remarkable differences were found between the use of tungsten versus gold particles in nopal. Gold particles were more effective in terms of overall foci production, leading to a statistically significant difference (*α* = 0.05) between these treatments. Therefore, thereafter, all the remaining analyses were done with gold particles (Table 1; Fig. 2d). Finally, we evaluated different osmotic agents. Seven different media were analyzed at a constant concentration of 0.2 M. We found statistically significant differences among all the analyzed treatments (*α* = 0.05) according to the Duncan test. The best treatment in terms of *foci* formation was mannitol at 0.2 M. Remarkable differences were found with mannitol 0.2 M compared to the rest of the treatments. However, sorbitol also gave acceptable foci levels (Table 1; Fig. 2e). Surprisingly, we did not find a synergic effect between all the combinations of osmotic agents analyzed that could enhance the *uidA* transient expression. This was particularly interesting for the mannitol + sorbitol treatment, since they did generate high *foci* formation levels per explants, on their own, but, when we combined them, we did not observe a positive effect. No positive GUS expression was observed in nopal untransformed explants (Fig. 2f), confirming that this plant does not possess endogenous GUS production.

#### Transient expression

**Fig. 3:**
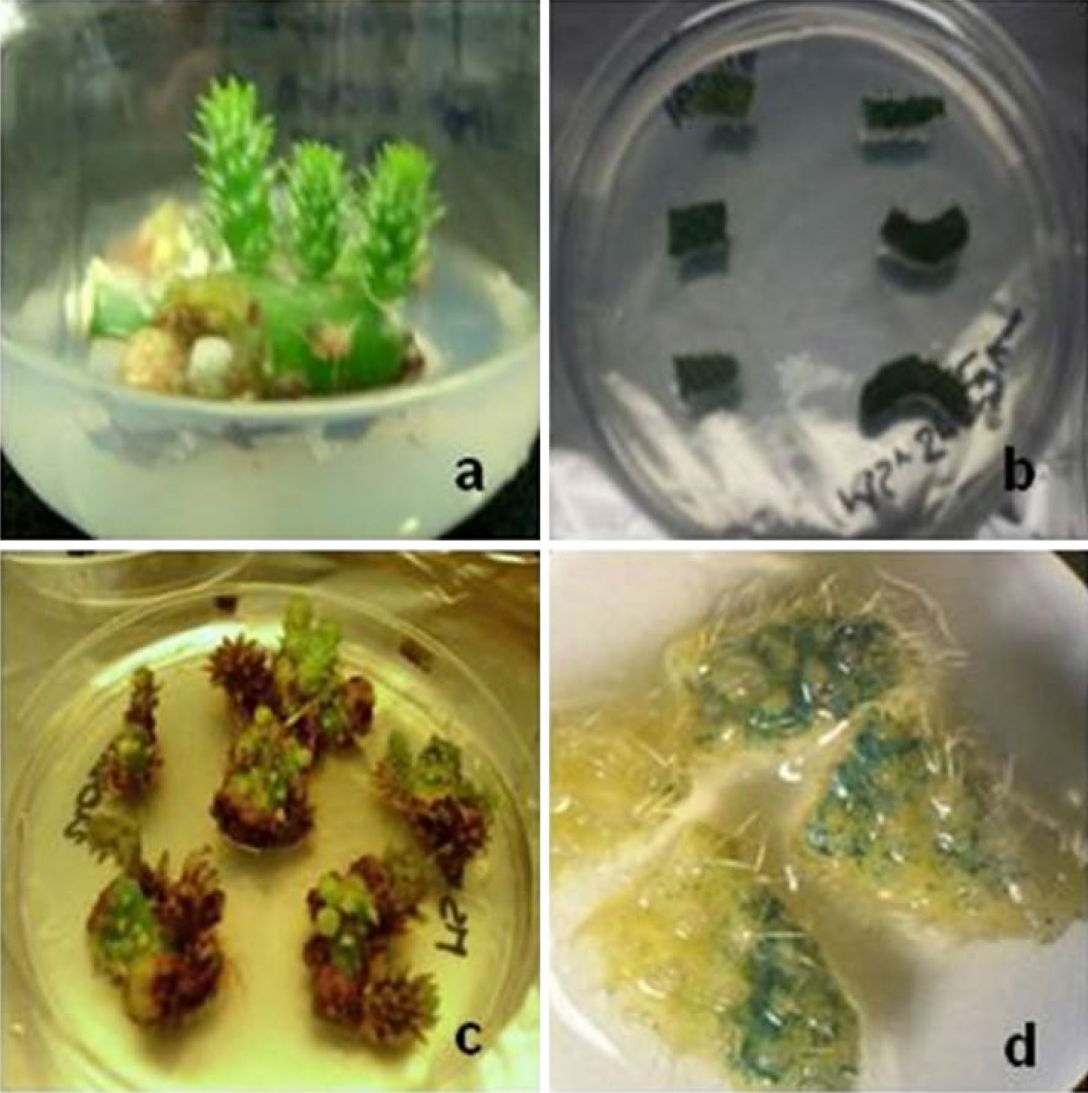
Nopal biolistic transformation. **a** Negative controls after particle bombardment with unloaded gold particles, **b** nopal explants after particle bombardment, (**c**) nopal transformed explants after 45 days in selection medium (100 mg/L kanamycin), **d** *uidA* transient expression in transformed nopal explants

With the conditions generated by transient expression, we evaluated stable gene integration in nopal explants. To determine the effect of our conditions on nopal cell damage and recovery after the biolistics process and to discard a low regeneration frequency due to mechanical damage, we made some shootings with unloaded gold particles (negative controls) in 1 cm^2^ nopal explants and also in *N. tabacum* var. Xanthi leaf sections. No statistical differences were found among the bombarded explants with respect to untreated nopal explants growing on regeneration medium (Fig. 3a). Thereby, we proceeded to our first stable transformation assay; 300 explants were bombarded, which were transferred to regeneration/selection medium (Fig. 3b, c). These explants were kept in this medium for 45 days, doing periodic transfers to fresh selection medium every 15 days to avoid antibiotic degradation which leads to false positives. During this time, we observed that non-transformed explants showed a high oxidation level and no regeneration. Overall transformation efficiency in terms of new bud formation and healthy appearance resulted in a 23% level of regeneration efficiency. Transient GUS expression was evident after particle bombardment events (Fig. 3d).

#### Stable integration of genes in nopal explants

**Fig. 4:**
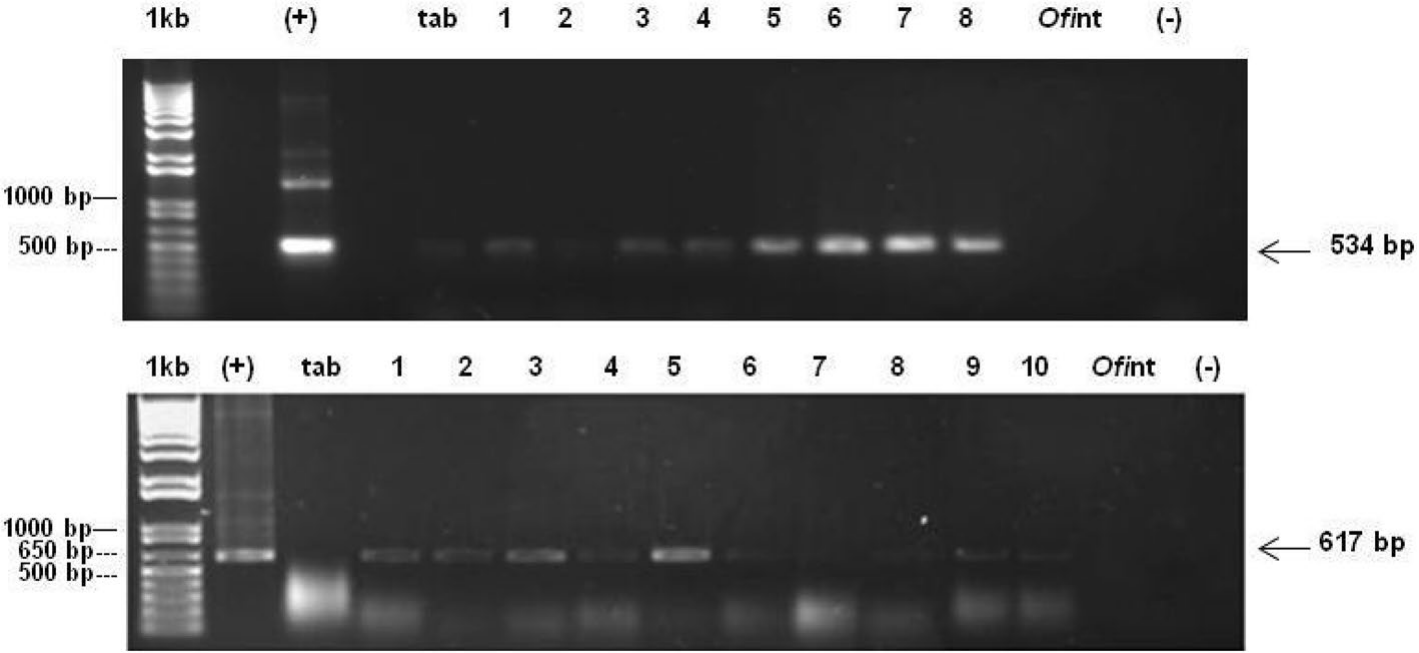
PCR amplification of the *nptII* and *uidA* transgenes in transformed nopal explants. **a** 534 bp fragment of the *uidA* gene, **b** 617 bp fragment of the *nptII* gene. (+), pBI426 (positive control), and transformed nopal samples (lanes 1 to 10), *Ofi*nt, untransformed nopal samples, tab, transformed *N. tabacum,* (−) deionized sterile water. 1 kb molecular weight marker

**Fig. 5:**
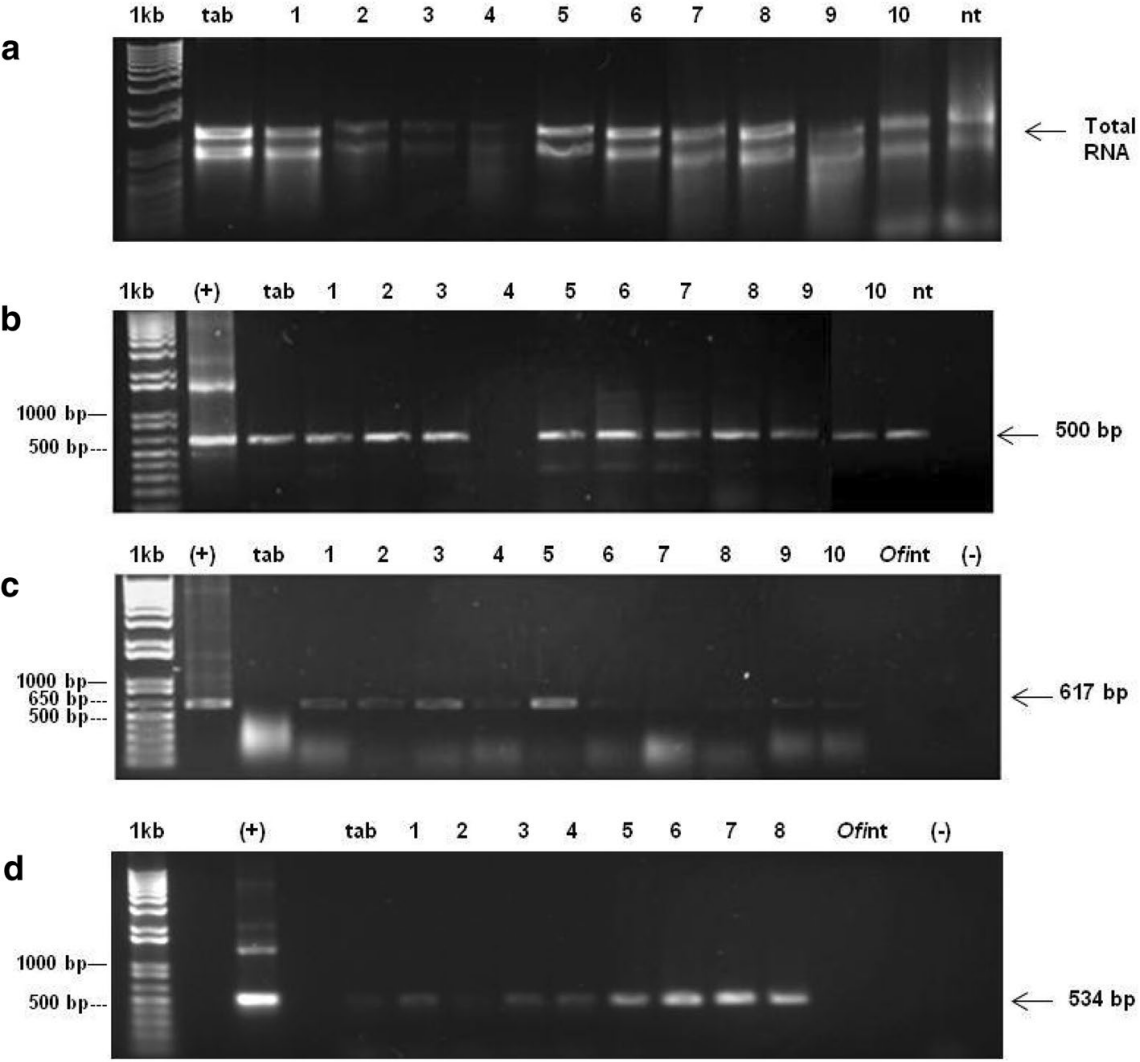
RT-PCR detection of both (*nptII* and *uidA*) genes in transformed nopal explants. **a** Total RNA, tab: *N. tabacum*, transformed nopal samples (lanes 1–10), and nt: untransformed nopal, **b** c-DNA from different samples, (+) actin gene tab: *N. tabacum* c-DNA, c-DNA from transformed nopal samples (lanes 1–10), nt untransformed nopal, c-DNA, **c** 617 bp fragment amplification of the *nptII* gene, (+) pBI426 plasmid DNA (positive control), tab: tobacco c-DNA, transformed nopal samples (lanes 1–10), *Ofi*nt: untransformed nopal sample, **d** 534 bp fragment amplification of the *uidA* gene, (+) pBI426 plasmid DNA, transformed nopal samples (lanes 1– 8), *Ofi*nt: untransformed nopal sample. 1 kb: molecular weight marker. (−) deionized sterile water

Nopal explants were transformed by the biolistic process as analyzed by means of PCR. A 617 bp fragment of the *nptII* gene located between the 1606 and 2221 pb of the pBI426 plasmid was detected in seven out of ten samples analyzed (Fig. 4a). Moreover, a 534 bp fragment of the *uidA* gene was also detected among the entire samples (Fig. 4b**)**. To effectively demonstrate the expression of both transgenes in the transformed nopal explants, we proceeded to the RT-PCR analysis. Total RNA was isolated from ten samples of putative transformed nopal explants, untransformed nopal explants (negative control), and transformed *N. tabacum* var Xanthi leaf sections (Fig. 5a**)**. These RNA samples were utilized to synthesize complementary DNA and to validate its quality; we amplified a 500 bp fragment for the actin gene in all samples (Fig. 5b**)**. We were capable of detecting the presence of the *nptII* gene in the ten samples (Fig. 5c**)**. Interestingly, the presence of the *uidA* gene was detected in only eight out of ten samples (Fig. 5d). To analyze the expression of the β-glucuronidase protein in transformed nopal explants, we conducted a GUS fluorescent assay in ten nopal transformed explants as well as samples of untransformed nopal explants (negative control).

The GUS values reported here are the results of four repetitions in all samples. In general, GUS values were in average 30 4 MU/min/mg of protein for the transformed explants, which were really low when compared to our positive control (β-glucuronidase) which exhibited 2447 4 MU/min/mg of protein. No significant statistical differences (*α* = 0.05) were observed among the activities found in the transformed explants, but a significant difference was observed in relation with the positive control and negative control (no expression was observed in these samples).

## Discussion

### Ideal conditions for particle bombardment in nopal

The genetic transformation of nopal is a very difficult process, as has been reported previously (Llamoca-Zarate et al. 1998, 1999a, b, Silos-Espino et al. 2006; Angulo-Bejarano 2013). In particular, genetic transformation by particle bombardment is marked by low efficiency of stably transformed cells, and thus, condition optimization is necessary. In addition, several conditions affect stable transformation in biolistics, namely: mechanical damage, toxicity, and the adjustment of pressure, distance of flight, and type of particles. A balance between minor damage caused by particle bombardment and a high number of cells transiently expressing the transgene is desirable (Chernobrovkina et al. 2007). Our results are not a product of optimization, but rather arise from the search for best particle bombardment conditions for nopal that pursuit high foci formation levels to ensure in part, a much higher number of stably transformed cells.

The previous reports have indicated the influence of helium pressure and target tissue distance on DNA entrance and tissue damage in plant cells (Rubio et al. 2004). In nopal, high-pressure systems using 1200 psi and 7.5 cm in callus and apical cultures led to satisfactory transient expression (Llamoca-Zarate et al. 1998, 1999b). However, higher transient expression levels were found in our investigation when applying a lower helium pressure **(**Table 1; Fig. 2a, b). In fact, at lower pressure, less tissue damage is inflicted (Petrillo et al. 2008). In addition, distance of flight has a very important role in tissue damage and DNA penetration in plants. In our study, when we increased the distance of flight, the GUS transient expression levels were considerably lower than the ones observed when using 8 and 11 cm (Table 1); this may be due to the velocity decrease of the microcarriers that reduces the penetration force and DNA integration (Suratman et al. 2010).

The osmotic treatment prior to particle bombardment is used to reduce cell damage, enhance transient GUS expression, increase stable transformation, and facilitate cell membrane stabilization after transformation (Suratman et al. 2010). The previous studies made in nopal utilized a 12–16-h period of exposition prior to particle bombardment, but the osmotic agent was not described (Llamoca-Zarate et al. 1999b). This differs consistently with our findings **(**Table 1**)**, since, when the exposition time was increased, we found a statistically significant decrease (*α* = 0.05) in *foci* production.

Transgene copy number is influenced by the amount of DNA used for every bombardment, and thus, single-copy integration is favored when lower amounts of DNA are loaded into microcarriers (Lowe et al. 2009). This is in agreement with our results. The highest GUS transient expression was observed when using 1.0 µg/µL (Table 1; Fig. 2c). This was also observed in plant species like tomato (Camacho-Beltrán 2008). Unfortunately, the previous reports in nopal particle bombardment do not describe which plasmid DNA concentration was used (Llamoca-Zarate et al. 1998, 1999b). On the other hand, limiting the DNA amount used for particle bombardment results in fewer DNA molecules covering an individual particle (Lowe et al. 2009). This coincides with our results, where the use of 0.5 µg/µL resulted in less foci production when compared with the use of 1.0 µg/µL. Interestingly, when a DNA concentration of 1.5 µg/µL was used, we did not observe a higher level of foci production **(**Table 1**)**.

Gold and tungsten particles differ in their ability to generate foci and stable transformation events due to their overall shape, penetration depth, toxicity, and foci formation size (Altpeter et al. 2005; Rivera et al. 2012). In the present study, gold particles gave a higher number of blue spots per explants (Table 1; Fig. 2d). The low levels of transient GUS expression in the tungsten bombarded explants could be due to a toxic effect (Rivera et al. 2012). Since nopal tissue architecture is more complex, this could prevent the penetration of tungsten particles into deeper cell layers, thereby generating a lower transient GUS expression level. Nevertheless, in the previous reports, tungsten particles of 1.3 µm diameter were used during the particle bombardment of nopal apical meristems, generating acceptable results in terms of transient GUS expression (Llamoca-Zarate et al. 1999b). Similar results to the ones reported here were registered in *Jatropha curcas*, where the use of 900 psi and 0.6 µm gold particles generated a higher survival percentage in bombarded tissues (Joshi et al. 2011).

The type and concentration of the osmotic agent may increase the transient gene expression by reducing the turgor pressure in cells, helping to increase the survival probability of the cells by avoiding cell rupture after the blast generated from the bombardment (Rosillo et al. 2003). The positive effect of using mannitol and combinations with sorbitol has already been reported in plant systems such as watermelon (Suratman et al. 2010) and coffee (Gatica et al. 2009). Even though the use of mannitol generated positive results in foci formation (Table 1), the sorbitol + mannitol treatment and the maltose + sorbitol + mannitol treatment did not generate any synergic effect that could enhance GUS expression. However, the results presented here are far higher than the ones reported by Llamoca-Zarate et al. (1999b). In other words, we report 197 positive GUS events (only for the mannitol 0.2 M treatment) versus 35 reported by that research group. In brief, the results presented here may be regarded as robust and allowed the establishment of nopal particle bombardment conditions by means of transient expression; such conditions are: a distance of flight to the target tissue of 8 cm; a helium pressure of 900 psi, a pre-conditioning period for the nopal explants of 4 h prior to the bombardment using 0.2 M mannitol as the osmotic agent, and the utilization of gold particles as microcarriers on which 1 µg/µL of plasmid DNA is previously loaded.

### Stable nopal genetic transformation by particle bombardment

Particle bombardment constitutes an alternative method for gene transfer in those cases where the other methods are not efficient, since it may facilitate DNA delivery into intact plant cells with no biological constraints or host limitations (Tassy et al. 2014; Wang et al. 2018). With our transformation protocol, we obtained a 23% of transformation efficiency and survival on selection medium which is higher than the ones reported for the other plant species such as marigold that was 1% (Vanegas-Espinoza et al. 2006), 8% for *A. tequilana* (Valenzuela-Sánchez 2006), and 1.8% for tomato (Camacho-Beltrán 2008). In addition, when we compared the results obtained with plant models more closely related to nopal, we found that the overall biolistic transformation efficiency in nopal was higher than the one previously registered for *Aloe vera* (8.7%) (Velchevá et al. 2010). Interestingly, our results are close to the ones reported for *Rhipsalidopsis gaertner* (22.7%) after *A. tumefaciens* genetic transformation (Al-Ramamneh et al. 2006).

In addition, Llamoca-Zarate et al. (1998, 1999b) reported transient GUS expression in nopal explants after particle bombardment; nevertheless, they never achieved stable genetic transformation or regeneration of the transformed explants. In addition, Cruz et al. (2009) reported 4% transformation efficiency by biolistics in nopal apical tissues. However, no stable integration of the transgene was demonstrated. Furthermore, Silos-Espino et al. (2006) achieved a 3.2% rate of genetic transformation efficiency in *O. ficus-indica* cv. Villanueva transformed by means of *A. tumefaciens*. However, the results presented here by the biolistics process are consistently higher. This could be due to the fact that the nopal transformation by the *A. tumefaciens* method presents some serious difficulties. Experiments to establish *O. ficus-indica* cv. Blanco sin Espinas genetic transformation by means of *A. tumefaciens* infection were conducted, with no positive results (data not shown). In fact, Karami et al. (2009) described that, when using the *A. tumefaciens*, gene transfer can be very different within the same plant species, even at the cultivar level. Therefore, the particle bombardment method established in nopal presents advantages, since one should not have to overcome plant-related issues such as those involved in the *A. tumefaciens* method.

### Transgene integration assessment in nopal stable transformants

Stable integration of the transgene into the plant genome by different genetic transformation protocols remains a very difficult task. One of the main disadvantages associated with the biolistics gene transfer method is related to the co-suppression phenomenon or transgene rearrangements that can prevent transgene integration, limit its proper expression, or inhibit the production of the foreign protein (Lowe et al. 2009). Thus, obtaining positive results in terms of transgene expression in nopal explants may be considered a very important step. With the method described previously, we were capable of detecting the integration of both *nptII* and *uidA* transgenes by means of PCR, in all the transformed samples (Fig. 3a, b). Other reports made in nopal with biolistics were able to detect only the transient GUS expression without demonstrating the stable integration of the transgene into the plant genome (Llamoca-Zarate et al. 1998, 1999b). However, the sole report that had previously demonstrated the capacity of nopal for the introduction of transgenes was published by Silos-Espino et al. (2006). In this case, the stable integration of the *nptII* gene was demonstrated by PCR and Southern blot hybridization. On the other hand, our method allowed the expression of these transgene transcripts as revealed by the RT-PCR analysis of transformed materials (Fig. 4c, d), therefore demonstrating the capacity of this plant to express heterologous transgenes. However, this happened in a slightly unexpected way, as 10 out of 10 samples revealed the expression of the *ntpII* transgene, while only 8 out of 10 revealed the expression of the *uidA* gene. No information is yet available for the nopal gene silencing machinery; however, we can deduct from the results obtained, that this behavior may be due to a co-suppression event that caused the “turning off” of the *uidA* transgene in this plant or maybe to transgene rearrangements during RNA transcription (Lowe et al. 2009).

Stable expression and production of proteins in nopal was analyzed by a GUS fluorescent assay. The values obtained in this report were in average 30 4MU/min/mg protein which were consistently lower than the ones detected in the control sample (commercial β-glucuronidase). Thus, this constitutes apparently the first report indicating the feasibility of using a very complicated plant for the introduction of foreign DNA into the plant genome and, in the last instance, the production of heterologous proteins.

## Conclusions

An efficient particle bombardment protocol for nopal (*O. ficus-indica*) cv. “Blanco sin Espinas” was developed; adjusting parameters for particle bombardment led to enhanced transformation efficiency as we have described in this study. Moreover, we were capable of detecting the *nptII* and *uidA* transgenes after the RT-PCR process, which demonstrates the capacity of nopal explants for the expression of heterologous genes. Finally, we were also capable of detecting adequate *β*-glucuronidase protein level, demonstrating that this ancient plant may be used for the production of heterologous proteins. Further studies are needed to improve the production of proteins in this plant, to use it as a bioreactor plant system.

## Abbreviations

2,4-D: Dichlorophenoxyacetic acid
BA: 6-bencilaminopurine
CAM: Crassulacean acid metabolism
CaMV35S: Cauliflower mosaic virus 35S constitutive promoter
GUS: β-glucuronidase enzyme
*nptII*: Gene coding for neomycin phosphotransferase II enzyme
*uidA*: Gene coding for β-glucuronidase enzyme
